# 

**Published:** 1898-12-10

**Authors:** 


**Notices of Births, Deaths, and Marriages** are inserted in this column at a charge of 2s. 6d. for an announcement not exceeding four lines.

### NOTICE TO CORRESPONDENTS.

All MS., Letters, Books for Review, and other matters intended for the Editor should be addressed The Editor, "The Hospital," The Hospital Building, 28 & 29, Southampton Street, Strand, W.C.

The Editor cannot undertake to return rejected MS., even when accompanied by stamped directed envelope.

**Notice to Advertisers and Subscribers.**

All Advertisements, Orders for Copies of The Hospital, and Business Communications should be addressed to **THE MANAGER (Not to the Editor),** The Hospital, The Hospital Building, 28 & 29, Southampton Street, Strand, London, W.C.

The Cost of Subscription, post free, is as follows:—Per week, 2½d.; per quarter, 2s. 8d.; per half-year, 5s. 3d.; per annum, 10s. 6d. Foreign:—Per annum, 15s. 2d., payable, in all cases, in advance.

**NOTICE.—Vol. XXIV. of "The Hospital" (April, 1898, to September, 1898), in handsome cloth binding, is now on sale at the Office of this Journal, price 7s. 6d. Cases for binding the Numbers contained in this Volume 1s. 6d. Index to Vol. XXIV., 2½d., post free.**

**The Monthly Part for December is now ready. Price 6d., by post 8d.**

### Scraps and Gleanings.

Lord Kitchener of Khartoum has become a vice-president of the Bethnal Green Free Library in place of the late Captain Sir Sydney Webb.

Plans of the recommended Isolation Hospital at Bedford have been forwarded to the Local Government Board and consent asked to borrow £6,500.

Last year, out of the total income of the Stockton Hospital of £2,492, nearly £1,850 was subscribed by the working men of Stockton and Thornaby.

Last year the sum subscribed by the 167,000 out-patients who attended the London Hospital was only £350, which is less than one halfpenny each.

The Convalescent Hospital on Cannock Chase raised to the memory of Sister Dora is now in danger of being closed in consequence of lack of funds. A sum of from £500 to £600 a year is required to carry it on, and it is hoped that this amount will be forthcoming and so obviate the closing of a useful institution which has for so long done such good work for the poor. Miss Lonsdale, who has carried on the institution for the past 15 years, has obtained promises of £200 a year providing another £200 is forthcoming by Christmas, but even then £400 will not be sufficient, and if an adequate sum is not obtained the home will have to be carried on as an institution for paying patients.

During the past 55 years the National Refuges for Homeless and Destitute Children have benefited over 14,500 poor boys and girls, and from the society's training ships "Arethusa" and "Chichester" over 6,000 lads have been sent into the merchant service and Royal Navy.

The building of the Chippenham Cottage Hospital, erected in commemoration of the Queen's Diamond Jubilee, is now completed. A management committee has been appointed with power to purchase the furniture, to select the officers for the institution, and to draw up the rules and regulations for the conduct of the hospital.

Lady Elizabeth Knox is appealing to the public on behalf of the Cripples' Nursery, which was opened in 1862 for about 50 boys and girls from three to twelve suffering from spinal complaints, infant paralysis, rickets, &c. The nursery is situated opposite Clarence Gate, Regent's Park, at 15, Park Place, and there is an airy garden attached to the house where the cripples can play. The children are paid for £14 a year each, but this is not nearly enough to cover the cost incurred.

The question of providing an isolation hospital in connection with the Battle Urban District Council has again been brought forward, and at a meeting of the Council held the other day it was resolved that this question be referred to the Sanitary Committee, with instructions to confer with their medical officer of health, and to bring up at an adjourned meeting definite proposals for the consideration of the Council.

The directors of the Perth Infirmary have before them the question of the desirability of extending the accommodation for fever patients. Increased accommodation for 20 more patients is required, and the city authorities and the County Council have proposed that the directors of the infirmary should provide it, and that they shall receive from the authorities a sum of £300 per annum for ten years. But the directors do not feel justified in going to this expense (about £3,000), and have thus intimated to the authorities. It now remains to be seen whether the City authorities will provide the necessary funds.

### The Student of Medicine.

### APPOINTMENTS.

J. F. Bill, M.B Lond., M.R.C.S.Eng., appointed Assistant Medical Officer to the Lewisham Union Infirmary. H. J. Butler, L.R.C.P.Edin., M.R.C S.Eng , appointed Medical Officer for the Seventh District of the Bradford (Yorks) Union. E. H. B. Fox, M.R.C.S.Eng., L.R.C.P.Lond., appointed House Surgeon to the Royal South Hants Infirmary, Southampton. A. C. Greenwood, M.R.C.S.Eng., appointed Medical Officer for the Burton Coggles District of the Grantham Union, and Medical Officer for the Corby District of the Bourne Union. W. Hern, M.R.C.S., L.D.S., appointed Lecturer in Dental Surgery and Pathology to the Dental Hospital of London. G. H. Higgins, M.R.C.S.Eng., L.R.C.P.Edin , appointed Medical Officer for the Third District of the Bradford (Yorks) Union. R. G. Hogarth, F.R.C.S.Eng., appointed House Surgeon to the Samaritan Hospital for Women, Nottingham. H. Horrocks, M.D., B.Sc.Lond., D.P.H., appointed Honorary Assistant Physician to the Public Hospital, Perth, Western Australia. P. MacLulich, B.A., M.B., B.Ch., B.A.O.-Dub.Univ., appointed Second Assistant Medical Officer to the Joint Counties Asylum, Carmarthen. J. Medcalfe, M.D.Brux., L.R.C.P., L.R.C.S.Edin., appointed Medical Officer for the Ninth District of the Bradford (Yorks) Union. J. A. Milne, M.B., appointed Assistant Medical Officer of the Leeds Union Workhouse. H. P. Noble, M.B., B.S.Lond., M.R.C.S., L.R.C.P., appointed Anæsthetist to the Metropolitan Hospital, Kingsland Road. W. R. Pirie, M B , C.M., A.M.Aberd., appointed Assistant Anæsthetist to the Royal Infirmary, Aberdeen. F. E. Ramsford, M.D., L.R.C.P., appointed Resident Medical Superintendent to the Stewart Institution, Palmerston, near Dublin. O. F. Rowley, M.R.C.S.Eng., L.R.C.P.Lond., appointed Honorary Surgeon to the Beckett Hospital and Dispensary, Barnsley. H. T. Sells, L.R.C.P.Edin., M.R.C.S.Eng , reappointed Medical Officer of Health to the Northfleet Urban District Council. T. Shennan, M.D.Edin., F.R C.S.Edin., appointed Pathologist to the Royal Hospital for Sick Children, Edinburgh. G. E. Scholefield, M.D.Edin., appointed Medical Officer of Health to the West Lancashire Rural District Council. M. K. Tebay, M.R.C.S.Eng., L.R C.P.Lond., appointed Assistant Medical Officer of the St. George-in-the-East Workhouse and Infirmary. H. de P. B. Veale, L.S.A , appointed Medical Officer for the Fourth District of the North Bierley Union. B. P. Viret, M.B.Lond., M.R.C.S.Eng., appointed Medical Officer for the Eighth District of the Bradford (Yorks) Union. H. J. Watson, M.D.Toronto, reappointed Clinical Assistant to the Chelsea Hospital for Women.

St. Thomas's Hospital.—The following gentlemen have been appointed House Officers from December 6th: House Physician, H. D. Singer, M.B.Lond., L.R.C.P., M.R.C.S. Obstetric House Physicians (Senior), J. F. McClean, L.R.C.P., M.R.C.S ; (Junior) R. H. Bell, B.A., M.B., B.C.Camb., L.R.C.P., M.R.C.S. Clinical Assistants in the Special Departments for Diseases of the Throat, Skin, and Ear.—Throat: H. E. Hewitt, M.B Lond., L.R.C.P., M.R.C.S.; W. C. Ambrose, B.A.Camb., L.R.C.P., M.R C.S. Skin: H. M. Scaping, B.A.Camo., L.R.C.P., M.R.C.S. Ear: H. T. Acland, L.R.C.P., M.R.C.S. Clinical Assistant in the X-ray Department: G. J. Arnold, F.R.C.S., L.R C.P. Clinical Assistants in the Electrical Department: E. A. Gates, L.R.C.P., M.R.C.S.; H. N. Goode, L.R.C.P., M.R.C.S.

Crown 8vo., Paper Covers, 6d.

**THE OPEN-AIR TREATMENT OF PHTHISIS.**
**A Visit to Falkenstein.**
By RICHARD GREENE, F.R.C.P.Ed.

London: The Scientific Press (Ltd.), 28 & 29, Southampton Street, Strand, London, W.C.

